# A Cell-Based Therapeutic Strategy for Stress Urinary Incontinence: Functional and Molecular Evidence from Decidua-Derived Mesenchymal Stromal Cells

**DOI:** 10.3390/ijms27156845

**Published:** 2026-07-30

**Authors:** Paz de la Torre, Jennifer Collado, Mª José Morán-Jiménez, Laura Forcén, Ana R. Masero-Casasola, Alicia García, Mª Carmen Gutiérrez-Vélez, José Medina-Polo, Eloy Muñoz, José Joaquín Merino, Ana I. Flores

**Affiliations:** 1 Grupo de Medicina Regenerativa, Instituto de Investigación Sanitaria Hospital 12 de Octubre (imas12), 28041 Madrid, Spain; torre-merino.paz@h12o.es (P.d.l.T.); jennifercollado.imas12@h12o.es (J.C.); moranjimenez@h12o.es (M.J.M.-J.); laura.forcen@salud.madrid.org (L.F.); anarrosa@yahoo.es (A.R.M.-C.); alixgarcia83@yahoo.es (A.G.); menchu_guti@hotmail.com (M.C.G.-V.); eloymgalligo@yahoo.es (E.M.); josejmerino.imas12@h12o.es (J.J.M.); 2 Servicio de Obstetricia y Ginecología, Hospital Universitario 12 de Octubre, 28041 Madrid, Spain; 3 Grupo de Salud Integral Masculina, Instituto de Investigación Hospital 12 de Octubre (imas12) y Servicio de Urología, Hospital Universitario 12 de Octubre, 28041 Madrid, Spain; jose.medina@salud.madrid.org; 4 Departamento de Farmacología, Farmacognosia y Botánica, Facultad de Farmacia, Universidad Complutense de Madrid (UCM), 28040 Madrid, Spain

**Keywords:** perinatal mesenchymal stromal cells, decidua-derived mesenchymal stromal cells (DMSCs), stress urinary incontinence (SUI), vaginal distension, urethral function, endopelvic fascia, tissue regeneration, regenerative medicine, fibroblast, senescence, extracellular matrix (ECM)

## Abstract

Stress urinary incontinence (SUI) is a highly prevalent condition associated with pelvic floor damage, fibroblast dysfunction, and impaired extracellular matrix (ECM) remodeling. This study aimed to investigate the regenerative potential and underlying molecular mechanisms of decidua-derived mesenchymal stromal cells (DMSCs) in a rat model of vaginal distension (VD) and in human suburethral fibroblasts from SUI patients. Adult female rats were subjected to VD and treated with periurethral DMSC injections, followed by functional and transcriptomic analyses, one and six weeks after VD. In parallel, an in vitro co-culture system was used to evaluate the paracrine effects of DMSCs on SUI fibroblasts. DMSC treatment significantly increased leak point pressure (LPP) one week after VD, approaching values observed in control animals (19.8 ± 0.45 vs. 22.3 ± 2.08 mmHg, *p* = 0.09), while promoting transcriptional changes consistent with tissue repair. Gene expression analyses revealed transient increases in proliferative and inflammatory markers, followed by earlier normalization compared to untreated animals. In vitro, DMSCs reduced p16 expression, increased p21 and Klotho levels, and rebalanced ECM remodeling by decreasing MMP-1 and increasing MMP-2. These findings indicate that DMSCs promote a regenerative microenvironment by modulating senescence and ECM dynamics. Overall, DMSCs may represent a promising disease-modifying strategy for SUI by enhancing tissue repair and functional recovery.

## 1. Introduction

Stress urinary incontinence (SUI) is defined as the unintentional loss of urine following a physical effort due to a weakened pelvic floor or urethral sphincter muscles. SUI is a prevalent condition among women, affecting up to one in three of them, particularly following childbirth or as part of the aging process [1]. SUI has been demonstrated to result in a multitude of psychosocial consequences, including social isolation, diminished self-esteem, sexual dysfunction, and augmented financial strain. Implementing effective management regimens often requires a multifaceted approach, including physiotherapy to strengthen the pelvic floor muscles and, occasionally, surgical interventions. These measures are undertaken with the objective of restoring functionality and enhancing quality of life [2]. SUI is the most common type of female incontinence, with a prevalence that varies from 23% in Spain to over 40% in France (44%), Germany (41%), and the UK (42%) [3].

The endopelvic fascia is a complex, three-dimensional network of connective tissue that lines the pelvic walls and floor. This fascia plays a critical role in maintaining pelvic organ support, including the vagina, uterus, and bladder. It also serves as a conduit for nerves and blood vessels [4]. Fibroblasts are the predominant cellular element of this tissue and are responsible for the production and remodeling of the extracellular matrix (ECM), which is a complex network of proteins that provides structural support [5]. The endopelvic fascial ECM is organized into a fibrillar network rich in collagen and elastic fibers, embedded within a hydrated ground substance composed of proteoglycans and glycosaminoglycans (GAGs) [6,7]. Consistent with their established roles in connective tissues, collagen provides tensile strength, whereas elastic fibers confer elasticity and recoil, thereby supporting the biomechanical function of the pelvic connective tissue [8,9]. Proteoglycans and GAGs constitute the hydrated ground substance of the ECM, maintaining tissue hydration, viscoelasticity, and resistance to compressive forces while contributing to collagen fibrillogenesis and matrix organization [8,9,10]. Beyond their structural role, these non-fibrillar components regulate matrix biomechanics, growth factor bioavailability, mechanotransduction, and cell signaling, thereby modulating fibroblast activity, extracellular matrix remodeling, tissue homeostasis, and tissue repair [8,10]. Damage to the endopelvic fascia is the underlying cause of weakness. The absence of restorative remodeling by fibroblasts leads to the sustained support of the pelvic organs [11].

Vaginal distension (VD) models are primarily used in preclinical animal studies to simulate pelvic floor trauma related to childbirth and SUI. The VD model has been reported to cause external urethral sphincter dysfunction and various physiological changes, including genitourinary organ hypoxia [12], loss of external urethral sphincter function [13], and reduced leakage pressure [14]. In response to VD, affected tissues activate cellular and molecular mechanisms to restore homeostasis. Animals subjected to a VD procedure typically show partial recovery of urethral function over time [15]. The time required for spontaneous recovery in the VD model has been reported to be directly proportional to the duration of distension. While functional improvement may occur, some structural effects of VD can persist for up to six weeks after a four-hour procedure in rats [16], and up to 12 weeks in ovariectomized rats [17]. The most significant biochemical changes associated with tissue repair following VD have been observed in the urethra compared to neighboring organs such as the bladder, vagina, and rectum, at least immediately after VD. This may be attributed to the urethra’s limited physiological exposure to large strains and its relatively less redundant vascular supply compared with surrounding pelvic organs. Consequently, the urethra may be more susceptible to injury resulting from excessive mechanical deformation and transient ischemic stress during vaginal distension [18].

Regenerative medicine, defined as the use of stem cells to repair or revitalize damaged tissues, has emerged as a promising therapeutic approach for stress urinary incontinence (SUI). Among the various cell types that have been studied, mesenchymal stromalcells (MSCs)—a population of adult stromal cells—represent one of the most promising and extensively investigated sources for stem cell–based research and therapy [19,20]. MSCs can be obtained from several tissues, including bone marrow, adipose tissue, dental pulp, and various perinatal tissues, such as the placenta, Wharton’s jelly, the amniotic membrane, the decidua, and others. Perinatal tissues have emerged as particularly promising sources, as they are typically discarded after childbirth, making them readily available, easy to obtain, and free from ethical concerns. Among these, cells derived from the maternal portion of the placenta (the decidua) have been identified as mesenchymal in nature and are known as decidua-derived mesenchymal stromal cells (DMSCs) [21]. DMSCs are a relatively homogeneous population of mesenchymal cells that have demonstrated therapeutic potential in various disease models, both in vivo and in vitro. This potential is largely attributable to their migratory behavior, antitumor effects, low immunogenicity, and immunomodulatory properties.

In a previous study conducted by our laboratory, we demonstrated that, six weeks after VD, functional recovery was significantly greater in animals treated with two injections of DMSCs than in untreated animals [14]. Histological analysis of the urethras of these animals revealed an improvement in tissue architecture following treatment with DMSCs, including greater integrity of the muscle fibers of the external urethral sphincter, reduced ECM infiltration, and better organization of the elastic fibers. Together, these findings suggested that DMSCs not only improved continence recovery but also modulated structural tissue remodeling.

The present study builds upon those findings by providing a molecular characterization of the regenerative response. Specifically, we performed transcriptomic analyses on urethral tissues collected from the same animals included in our previous publication in order to identify the molecular pathways associated with the functional and histological improvements observed at six weeks. Rather than reassessing tissue morphology, which has already been comprehensively described, this work was designed to investigate the biological mechanisms through which DMSCs may modulate tissue repair and ECM remodeling following VD. To this end, we analyzed the expression of genes involved in apoptosis (*Bax/Bcl-2*), cell proliferation (*Ki67*), angiogenesis (*Vegf* and *Fgf*), ECM remodeling (*Tgf-β, Acta2* (α-SMA)*, MMP-1, MMP-2* and *TIMP-1*), inflammation (*Ccl2* and *Ccl7*), and cellular senescence (*Klotho* and gene encoding p16).

As most changes caused by VD become evident shortly after injury, we also included an independent 1-week cohort to characterize the early transcriptional response to DMSC treatment. The combination of this early molecular analysis with the late molecular characterization of the previously described cohort provides a temporal perspective on the biological processes associated with DMSC-mediated repair. By integrating molecular findings with the previously reported functional and histological outcomes, this study offers new mechanistic insight into the temporal regulation of urethral repair following childbirth-related injury.

## 2. Results and Discussion

### 2.1. The Effect of DMSCs on Urodynamic Parameters and on a Stress Urinary Incontinence Model in Rats Induced by Vaginal Distension

**Figure 1:**
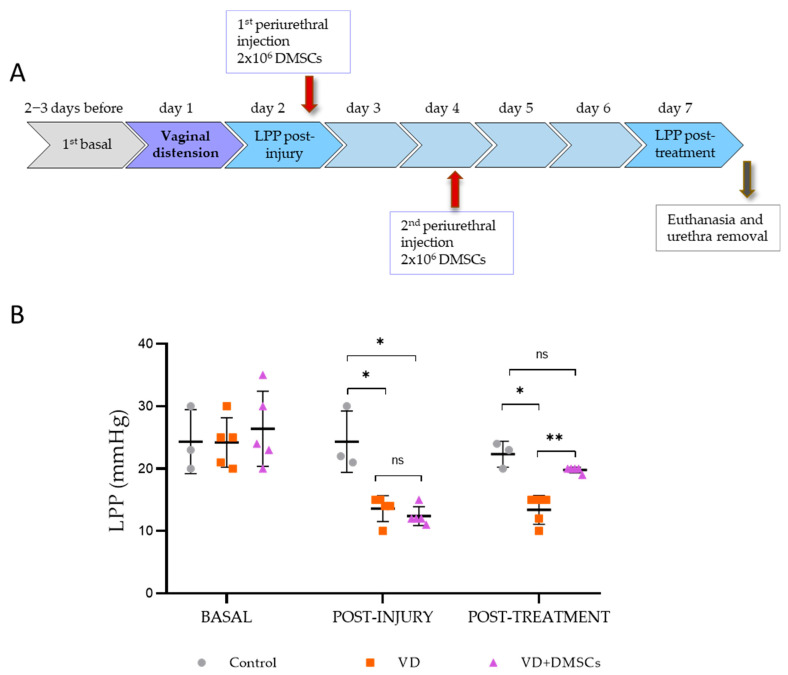
Periurethral injection of DMSCs significantly improved continence in rats one week after vaginal distension (VD). (**A**) Flowchart of the design for leakage point pressure (LPP) measurements, vaginal distension, and DMSC treatment. The diagram illustrates the interventions in the VD model, including measurements of the LPP (at baseline, after injury, and after treatment) and VD. Red arrows indicate DMSC treatments and a gray arrow indicates the end of the experiment, which includes animal euthanasia and tissue collection. (**B**) Leak point pressure (LPP) was measured in animals before and after VD and treatment with DMSCs (VD + DMSCs) or a saline solution (VD). “Basal” refers to the LPP measurements taken during the two days prior to VD. “Post-injury” refers to the LPP measurement taken one day after VD and before the injection of DMSCs. “Post-treatment” refers to the LPP measurements taken one week after injury, and following two injections of DMSCs or saline. A significant decrease in LPP was observed in the saline group (VD) and in the DMSC treated group (VD + DMSCs) one day after VD (post-injury). DMSC injection restores normal LPP values in comparison with rats that have been injected with saline (post-treatment). One week after the procedure, the incidence of incontinence was comparable between the DMSC group and the control group. Data are presented as the mean ± standard deviation (SD). * *p* < 0.05; ** *p* < 0.01; ns, not significant.

We developed two procedures to study the therapeutic use of DMSCs in adult, virgin Sprague-Dawley rats that were subjected to vaginal distension (VD). The first procedure, which has been reported previously, evaluated the functional and histological recovery of the urethra six weeks after injury [14]. Here, we present the results obtained using the second procedure, where the recovery was evaluated one week after injury (Figure 1A). Baseline leak point pressure (LPP) values were determined two or three days before vaginal distension. Damage was assessed by LPP after VD and one-week post-procedure (see Figure 1A). The animals were divided into three groups prior to the experimental intervention: a control group (CONTROL) with no damage or treatment, an untreated group with VD but without cell treatment (VD), and a group with VD and cell treatment (VD + DMSCs). The flowchart of the design for LPP measurements, VD, and DMSC treatment at six weeks can be viewed in our previous publication [14]. LPP measurements were performed in a blinded manner, without knowledge of the group assignment of each animal, in order to minimize bias. The impact of VD on LPP was evident, with a significant decrease observed in both groups of animals compared to the control group (Figure 1B; post-injury). However, the results of the one-week measurement indicated that the administration of two doses of DMSC following injury led to a significant improvement in LPP in the treated group compared to the untreated group (Figure 1B; post-treatment).

### 2.2. Expression of Genes Related to the Urethral Repair Process

To characterize the molecular response to vaginal distension (VD) and DMSC treatment, we analyzed the expression of genes involved in tissue injury and repair in urethral samples obtained from both experimental procedures (one-week and six-week procedures). The urethral tissues that were analyzed at 6 weeks were obtained from animals in which our group had previously reported that treatment with DMSCs improved functional recovery and was associated with a restoration of tissue integrity [14]. We also included a separate 1-week cohort to characterize the early transcriptional response to DMSC treatment, with the aim of providing a temporal perspective on the biological processes associated with DMSC-mediated repair. The 1-week cohort included five animals in both the VD and VD + DMSC groups. The 6-week cohort included six animals in the VD + DMSC group and five in the VD group, as one urethral sample was excluded due to insufficient RNA quality for molecular analysis.

#### 2.2.1. Expression of Genes Related to Apoptosis and Proliferation

**Figure 2:**
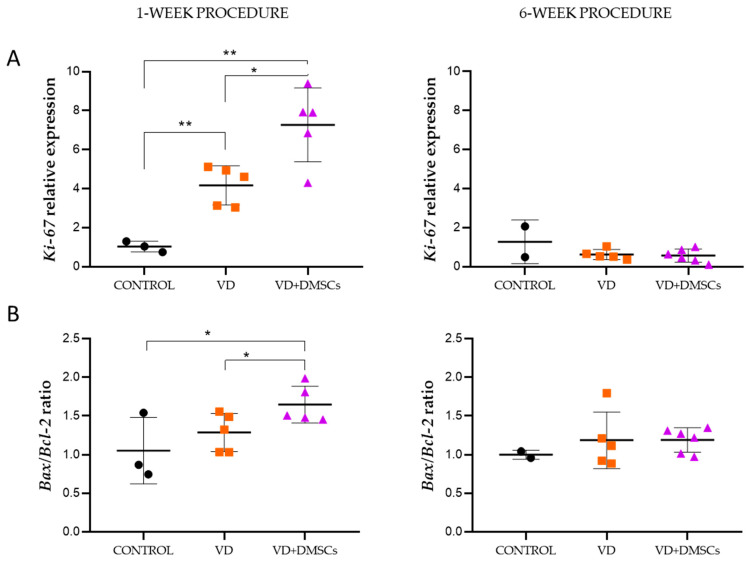
Expression of genes related to proliferation (*Ki67*) and apoptosis (*Bax* and *Bcl-2*) in rats one and six weeks after vaginal distension (VD). (**A**) *Ki67* relative expression (fold change vs. control), as determined by real-time qPCR. (**B**) Expression of *Bax* and *Bcl-2*, as determined by qPCR and expressed as the *Bax/Bcl-2* ratio. The genes were analyzed in animals that were either control (CONTROL), untreated (VD), or DMSC-treated (VD + DMSCs). Data are presented as the mean ± standard deviation (SD). * *p* < 0.05; ** *p* < 0.01.

First, we evaluated apoptosis and cell proliferation, as these processes allowed us to understand the dynamics of urethral tissue turnover. Apoptosis was evaluated using the *Bax/Bcl-2* expression ratio, while *Ki67* expression was evaluated as a marker of cell proliferation. One week after VD, a significant increase in *Ki67* expression was observed in both experimental groups compared to the control group. An increase in the *Bax/Bcl-2* ratio was also observed, though it was only significant in the urethras of the treated animals (Figure 2). The increase in these parameters one week after VD is consistent with the ongoing phase of tissue remodeling following injury. This phase is characterized by the controlled death of damaged cells (apoptosis) and an increased rate of cell proliferation to replace lost cells [22]. The changes observed in the animals treated with DMSCs (VD + DMSCs) were significant compared to those in the untreated animals. This suggests that the treated animals had a more potent regenerative response at this time.

To analyze the repair process over time, *Bax/Bcl-2* expression ratio and *Ki67* expression were examined in the six-week group. However, no significant differences were observed between the treated (VD + DMSCs) and untreated groups (VD) at this stage. The *Bax/Bcl-2* ratio approached baseline levels, indicating that the damage-induced apoptotic response had been resolved (Figure 2). Furthermore, *Ki67* expression decreased in the experimental groups by the sixth week. Evaluating these parameters alone at six weeks did not allow us to determine the extent to which tissue repair has been completed. However, the behavior of these parameters by the sixth week suggests that urethral tissue in both groups may have reached an advanced stage of repair and resolution. In conclusion, these findings suggest that DMSC treatment does not necessarily alter the final outcome. Rather, it enhances the magnitude of the initial repair response, which facilitates the overall repair process as seen at the functional level.

#### 2.2.2. Chemokine Gene Expression

Chemokines play a pivotal role in tissue repair [23]. These proteins regulate homeostasis and are produced by various cell types in response to tissue damage [24]. While their presence was initially associated with the onset of the inflammatory response, evidence suggests that they also play a significant role in the resolution phase of tissue damage [25]. By recruiting and guiding immune and progenitor cells to the site of injury, they coordinate the initial inflammatory phase and facilitate the transition to the remodeling stages [22,26].

**Figure 3:**
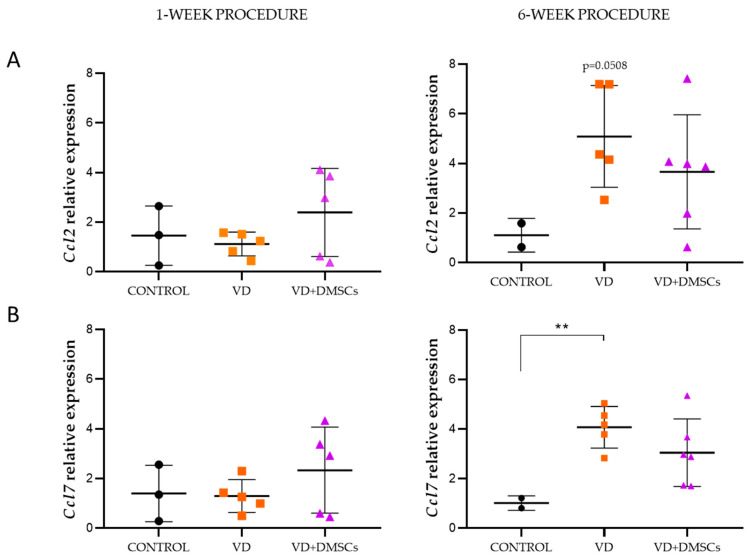
Expression of *Ccl2* and *Ccl7* in rats one or six weeks after vaginal distension (VD). (**A**) *Ccl2* relative gene expression (fold change vs. control), as determined by qPCR. (**B**) Relative gene expression of *Ccl7*, as determined by qPCR. The genes were analyzed in animals that were either control (CONTROL), untreated (VD), or DMSC-treated (VD + DMSCs). Data are presented as the mean ± standard deviation (SD). ** *p* < 0.01.

We focused on the chemokines CCL2 (MCP-1) and CCL7 (MCP-3) because they are both strongly upregulated in urethral and bladder tissues after ischemic, mechanical, or childbirth-related injuries [27]. In week 1, there were no significant differences observed between the two treatment groups (VD and VD + DMSCs) and the control group for either chemokine (Figure 3A,B). An increase in the levels of both *Ccl2* and *Ccl7* was observed in some animals in the DMSC-treated group (VD + DMSCs). This increase was not observed in the untreated group (VD). By week 6, the expression of *Ccl2* and *Ccl7* was above control levels in most animals, both treated (VD + DMSCs) and untreated (VD, Figure 3A,B). The increase was significant for *Ccl7* (** *p* < 0.01) but did not reach statistical significance for *Ccl2* (*p* = 0.0508) in the untreated (VD) group. In the treated group (VD + DMSCs), levels of both chemokines were higher compared to the control group, although the difference was not statistically significant. It is noteworthy that these levels were lower compared to the untreated group (VD), suggesting that the repair process was more advanced in the animals treated with DMSCs.

Studies by other authors have shown that *Ccl7* is rapidly upregulated in the urethral tissue of rats immediately after vaginal distension, and returns to baseline within 24 h [28]. In light of this, our data on *Ccl2* and *Ccl7* expression may suggest that the acute phase of inflammation has likely subsided in most animals after the one-week experimental procedure. The elevation observed at week six is more consistent with a secondary phase of chemokine activity associated with tissue remodeling. During this phase, chemokines contribute to the mobilization of progenitor cells, the deposition of the ECM, and angiogenesis, in addition to immune cell recruitment [25,29].

Both CCL2 and CCL7 are involved in the recruitment of mesenchymal stromal cells (MSCs) through the CCR2/CCL2 and CCR1/CCL7 signaling pathways [30,31,32,33,34,35]. Experimental models have shown that activating these pathways improves functional recovery and enhances stem cell homing after urogenital injury [32]. In this context, the increased expression of *Ccl2* and *Ccl7* by week 6 may indicate an active reparative microenvironment that promotes progenitor cell recruitment and tissue remodeling, rather than ongoing acute inflammation.

Importantly, chemokine signaling must be tightly regulated during the repair process. CCL2 and CCL7 are essential for effective regeneration; however, their sustained overexpression can promote chronic inflammation, fibrosis, and pathological remodeling. Insufficient expression, on the other hand, impairs macrophage recruitment and delays healing [23,25,36]. In our study, chemokine levels in several DMSC-treated animals approached control levels by week 6, whereas untreated animals maintained higher expression levels. These results imply that DMSC therapy could speed up the resolution of the inflammatory-reparative response and encourage a more efficient transition to tissue homeostasis [37].

#### 2.2.3. Growth Factors Associated with Regeneration

**Figure 4:**
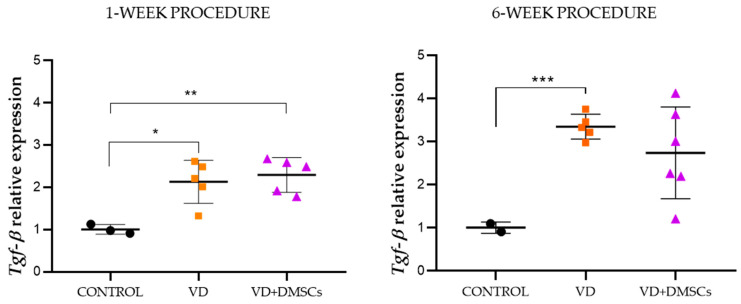
Expression of transforming growth factor-beta (*Tgf-β*) gene in rats, one and six weeks after vaginal distension (VD). The *Tgf-β* gene was analyzed by qPCR in the urethral tissue of animals that were either control (CONTROL), untreated (VD), or DMSC-treated (VD + DMSCs). *Tgf-β* relative gene expression (fold change vs. control) has been determined for each condition. Data are presented as the mean ± standard deviation (SD). * *p* < 0.05; ** *p* < 0.01; *** *p* < 0.001.

Transforming growth factor beta (TGF-β) plays a key role in maintaining homeostasis and promoting tissue renewal under physiological conditions [38]. In pathological conditions, TGF-β signaling plays a key role in regulating inflammation and wound healing [39]. TGF-β has been shown to stimulate collagen formation and extracellular matrix (ECM) synthesis while decreasing its degradation [40]. Dysregulation during the tissue repair process can lead to fibrosis or poor wound healing. In the present study, *Tgf-β* expression increased in both VD groups (VD and VD + DMSCs) at the week 1 procedure, reaching significantly higher levels than in the control group. By week 6, *Tgf-β* expression remained elevated in both groups compared to the control group. However, in the treated group (VD + DMSCs), *Tgf-β* levels remained at levels similar to those observed in week 1, while in the untreated animals (VD), they were higher (Figure 4).

The *Tgf-β* induction observed in week 1 in both groups (VD and VD + DMSCs) after injury is consistent with its role in activating the repair response, which requires the synthesis of ECM components. Subsequent attenuation of TGF-β is essential for maintaining collagen balance during injury recovery and preventing the excessive and disorganized accumulation of ECM, which could lead to fibrosis [41]. In fact, *Tgf-β* levels six weeks after VD in half of the DMSC-treated animals (VD + DMSCs) approached the levels observed in the control animals. Other authors have linked fibromuscular damage in the urethra with activation of the TGF-β/Smad pathway four weeks after postpartum vaginal dilation and bilateral ovariectomy [42]. At the six-week procedure, it was observed that the increase in *Tgf-β* had already ceased in the DMSC-treated group and was no longer rising, as the relative expression levels were closer to the control levels (Figure 4). It is evident that, in some of the animals within this group (VD + DMSCs), *Tgf-β* expression levels have converged towards those observed in the control group. This finding could suggest that some animals in the DMSC-treated group may be entering a subsequent remodeling phase. However, this remodeling phase has not yet occurred in the untreated group (VD), which at six weeks has higher *Tgf-β* levels than in the week one (Figure 4). This suggests that DMSC treatment (VD + DMSC) may restore the balance between ECM degradation and synthesis more rapidly, leading to enhanced remodeling compared to untreated animals (VD).

**Figure 5:**
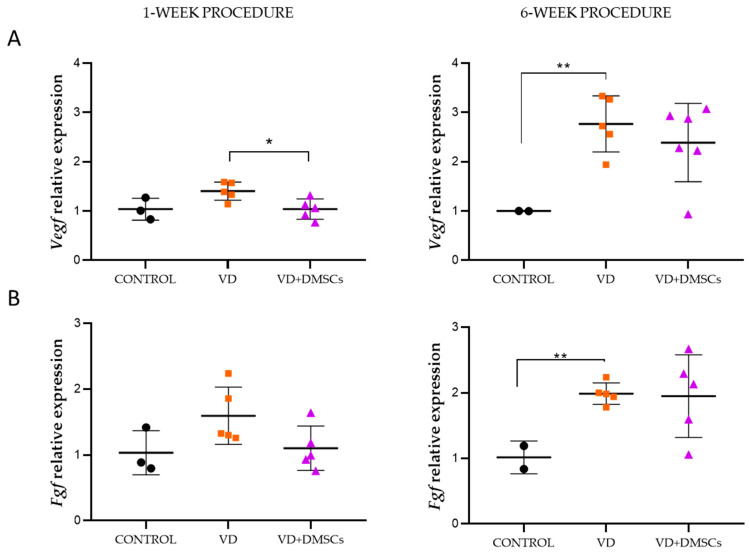
Expression of vascular endothelial growth factor (*Vegf*) and fibroblast growth factor (*Fgf*) genes in rats one or six weeks after vaginal distension (VD). (**A**) *Vegf* relative gene expression (fold change vs. control), as determined by qPCR. (**B**) Relative gene expression of *Fgf*, as determined by qPCR. The genes were analyzed in animals that were either control (CONTROL), untreated (VD), or DMSC-treated (VD + DMSCs). Data are presented as the mean ± standard deviation (SD). * *p* < 0.05; ** *p* < 0.01.

Vascular endothelial growth factor (VEGF) and fibroblast growth factor (FGF) are key proteins involved in tissue repair. Research indicates that they act synergistically [43]. VEGF is responsible for promoting the formation of new blood vessels that will perfuse the injured area, while FGF promotes cell proliferation, tissue regeneration, and modulates the inflammatory process [43,44]. In our study, an increase in both factors was observed in urethral tissue in response to VD. This early response was attenuated by DMSC treatment. At week 1, the mean expression level of these factors was approximately equivalent in DMSC-treated animals (VD + DMSCs) and controls, while untreated animals (VD) exhibited increased *Vegf* and *Fgf* expression (Figure 5). By week six, expression levels were higher than in the control group in both VD groups, though the increase was statistically significant only in the untreated animals (Figure 5).

As previously reported, experimental models of vaginal distension exhibit a spontaneous recovery process driven by endogenous repair mechanisms [15]. The increased expression of *Vegf* and *Fgf* observed in our study would be consistent with this reparative response. Persistent VEGF expression during the later stages of healing may be a reflection of ongoing tissue remodeling [45]. Because VD causes hypoxic injury, the timing of VEGF expression is particularly important. VEGF plays a dual role in tissue repair. In the acute phase, VEGF is upregulated, increasing vascular permeability and contributing to edema and inflammation. During the reparative phase, VEGF promotes angiogenesis and tissue regeneration [46]. Our findings suggest that DMSC treatment may modulate the temporal response of VEGF. The lower *Vegf* expression observed in DMSC-treated animals after one week may have limited the early increase in vascular permeability and tissue edema. This, in turn, may have contributed to the improved urethral function observed at this stage. Conversely, the increase in *Vegf* expression at six weeks likely reflects the activation of angiogenic pathways involved in tissue remodeling and regeneration, as previously described in hypoxic injury models [47]. However, VEGF activation should be transient. Persistent elevation may indicate incomplete healing. As demonstrated in animal models, the pharmacological inhibition of angiogenesis has been shown to improve healing outcomes [48]. At week 6, the gene expression levels of the cytokines VEGF and FGF remain above the control levels (Figure 5). However, in some animals treated with DMSCs (VD + DMSCs), these values are approaching that of the control group. This result has not yet been observed in the untreated (VD) group, suggesting that the animals treated with DMSCs appear to have a more rapid progression through repair process [49].

Successful tissue repair depends on the activation of multiple growth factors and cytokines, as well as their coordinated temporal expression. Previous studies have shown that wound healing improves when immunomodulatory signals, such as TGF-β, predominate in the early phase. This is followed by the sequential activation of pro-angiogenic and regenerative factors, including VEGF and FGF [50]. In untreated VD animals, *Tgf-β*, *Vegf*, and *Fgf* were induced early after injury. In contrast, DMSC-treated animals exhibited delayed increases in *Vegf* and *Fgf* at week 6, which coincided with declining *Tgf-β* expression in some animals. This temporal shift suggests that DMSC treatment may promote more coordinated progression through the phases of tissue repair.

**Figure 6:**
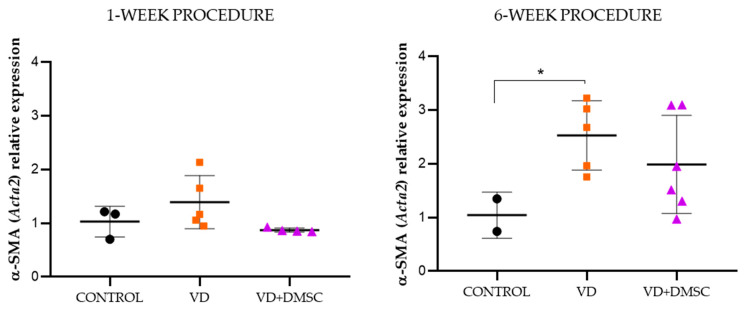
Expression of smooth muscle alpha-actin (α-SMA) gene (*Acta2*) in rats one or six weeks after vaginal distension (VD). *α*-SMA (*Acta2*) relative gene expression (fold change vs. control) was determined by qPCR. The gene was analyzed in animals that were either control (CONTROL), untreated (VD), or DMSC-treated (VD + DMSCs). Data are presented as the mean ± standard deviation (SD). * *p* < 0.05.

Smooth muscle alpha-actin (α-SMA) plays a critical role in the field of molecular biology. In addition to its function as a cytoskeletal protein associated with contractility, it is also a molecular indicator of tissue repair and remodeling. The expression of α-SMA can be altered by mechanical stress and hypoxia, as occurs after VD [51]. A slight early increase in *α*-SMA (*Acta2*) expression was observed in several of the untreated (VD) animals, which persisted and was significant at six weeks (Figure 6). In contrast, DMSC-treated (VD + DMSCs) animals exhibited a delayed induction of *α*-SMA (*Acta2*), with lower expression levels even at 6 weeks compared to untreated animals (VD) (Figure 6).

In the urethra, *α*-SMA (*Acta2*) expression can originate from both resident smooth muscle cells and activated myofibroblasts involved in tissue remodeling after injury [52]. An increase in *α*-SMA (*Acta2*) expression could indicate improved smooth muscle restoration, but it could also indicate profibrotic remodeling, highlighting the dual role of α-SMA during urethral repair [53]. According to the literature on fibrotic disease models in the urinary and genital tracts, tissue injury promotes a reparative response, in which TGF-β stimulates the activation and differentiation of resident fibroblasts into α-SMA-expressing myofibroblasts. This results in increased ECM deposition and tissue contraction [54]. While this response may initially contribute to structural repair, persistent activation of the TGF-β/myofibroblast axis and sustained α-SMA expression can drive pathological fibrosis [55]. As with the other processes studied in this work, ensuring adequate resolution and self-limitation of the TGF-β/α-SMA response is essential to prevent the loss of normal tissue. In this context, DMSCs may contribute to fine-tuning this response, promoting timely attenuation of profibrotic signaling and favoring proper tissue remodeling.

In conclusion, our findings in the animal model of incontinence mediated by VD demonstrate the efficacy of DMSCs in promoting functional recovery. As outlined in our previous publication, DMSC administration enhances the structure of urethral tissue [14]. The present study suggests that DMSC treatment may promote the earlier resolution of injury-associated molecular pathways in urethral repair following VD. In the long-term study (six weeks), the untreated group (VD) demonstrated significant and sustained dysregulation of most markers associated with injury and remodeling. Increased levels of α-SMA and TGF-β could also contribute to abnormal ECM remodeling six weeks after VD induction in this group. However, in the group treated with DMSCs (VD + DMSCs), a tendency to return to normal levels was observed in some animals. These findings suggest that DMSCs may promote proper and controlled remodeling of ECM components, reducing the risk of developing fibrosis.

Although fibrosis was not directly evaluated in the present study, the urethral tissues analyzed here at 6 weeks were obtained from the same experimental cohort previously described by our group [14]. In that study, histological analyses demonstrated improved tissue organization, preservation of striated muscle architecture, and a reduction in collagen deposition following treatment with DMSCs [14]. Therefore, the transcriptional changes observed in genes involved in ECM remodeling are consistent with the histological findings and provide an additional mechanistic perspective on the molecular processes associated with DMSC-mediated tissue repair. The use of the same cohort at 6 weeks in both studies eliminates inter-experimental variability and allows for the establishment of a direct correlation between functional, histological, and molecular findings in the same individuals. Furthermore, animals from a new cohort analyzed at one week were used to study the temporal mechanism of the biological effect of DMSCs on functional and tissue repair. However, it is important to note that these gene expression data should not be interpreted as direct evidence of reduced fibrosis. To further validate these mechanisms, future studies integrating molecular and histological analyses within the same experimental framework are necessary.

### 2.3. The Paracrine Effects of DMSCs on Suburethral Fibroblasts Isolated from Incontinent Women

In our previous work, we thoroughly characterized suburethral fibroblasts isolated from patients with stress urinary incontinence (SUI) [14]. These SUI fibroblasts were found to have reduced proliferative capacity and increased senescence markers when compared to fibroblasts from women without incontinence. Cellular senescence is a pro-inflammatory condition closely associated with defective tissue remodeling [56]. A notable increase in cellular senescence markers has been observed in SUI tissues [57]. Based on these observations, we hypothesized that DMSC injection could promote a rejuvenating or senescence-modulating effect on damaged urethral tissue in animals following VD [14]. Such an effect could contribute to the improved urethral function (LPP) previously observed after DMSC treatment [14], and may also explain the trend toward normalization of several biochemical markers reported in the present study. To test this hypothesis, we established an in vitro co-culture model using fibroblasts from women with SUI and DMSCs. We then evaluated the expression of senescence-associated markers in fibroblasts cultured in the presence or absence of DMSCs, focusing on pathways involved in cell cycle arrest, ECM remodeling, and tissue homeostasis.

**Figure 7:**
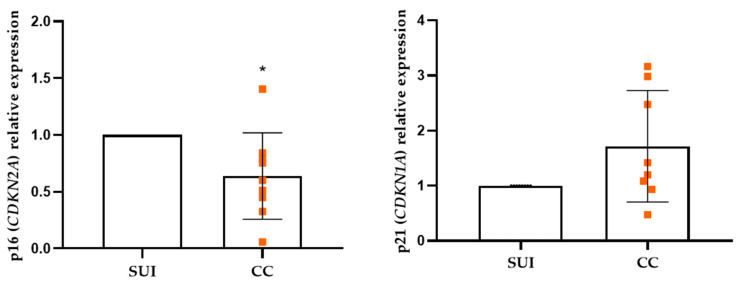
Analysis of senescence markers in SUI fibroblasts after co-culture with DMSCs. Paracrine effect of DMSCs on SUI fibroblast co-cultured with on Transwell^®^ culture inserts. Fold changes in p16 (*CDKN2A*) and p21 (*CDKN1A*) in SUI fibroblasts co-cultured with DMSC (CC) in relation to non-co-cultured SUI fibroblasts (SUI) (*n* = 8). Data are presented as the mean ± standard deviation (SD). * *p* < 0.05.

The expression levels of the genes encoding p16 (*CDKN2A*) and p21 (*CDKN1A*) two key regulators of cell cycle, were evaluated. These genes encode two proteins that are activated in response to DNA damage, oxidative stress, and other cellular stressors. In the pathophysiology of pelvic floor disorders, particularly pelvic organ prolapse, and stress urinary incontinence (SUI), hypoxia [58], and mechanical overload [59] are two convergent stimuli capable of inducing cellular senescence by activating the p16 and p21 pathways [57]. As illustrated in Figure 7, the co-culture of SUI fibroblasts with DMSCs led to a significant reduction in p16 (*CDKN2A*) expression, while p21 (CDKN1A) expression levels exhibited an increase in most co-culture samples. These findings suggest that DMSCs may be exerting only a partial modulatory effect on SUI cell senescence, and/or that the effects of DMSCs on senescence are independent of p21. Although p16 and p21 are both well-established markers of senescence, they are not functionally equivalent. As such, they may indicate different biological conditions [60]. Published reports generally associate p16-related senescence with a persistent, stable, and difficult-to-reverse arrest of cellular proliferative capacity. In contrast, signaling via p21 has been described as a more dynamic and potentially reversible process [61,62]. In addition to its well-established role in cell cycle arrest, p21 has been shown to contribute to skin wound healing due to its ability to regulate cell migration, differentiation, and ECM remodeling [63]. Furthermore, some of the beneficial effects of senescence on tissue formation are mediated by p21, which modulates ECM-associated genes and facilitates the synthesis of a functional ECM [64]. Therefore, DMSCs would modulate SUI cell senescence in co-culture by attenuating the more persistent senescent phenotype and promoting a transient stress response program potentially linked to tissue remodeling and repair. It is important to note that transcriptional changes in p16 and p21 alone are not sufficient to determine changes in the senescence state. Future studies will require complementary assays, such as SA-β-gal activity, DNA damage markers, and protein-level analysis.

**Figure 8:**
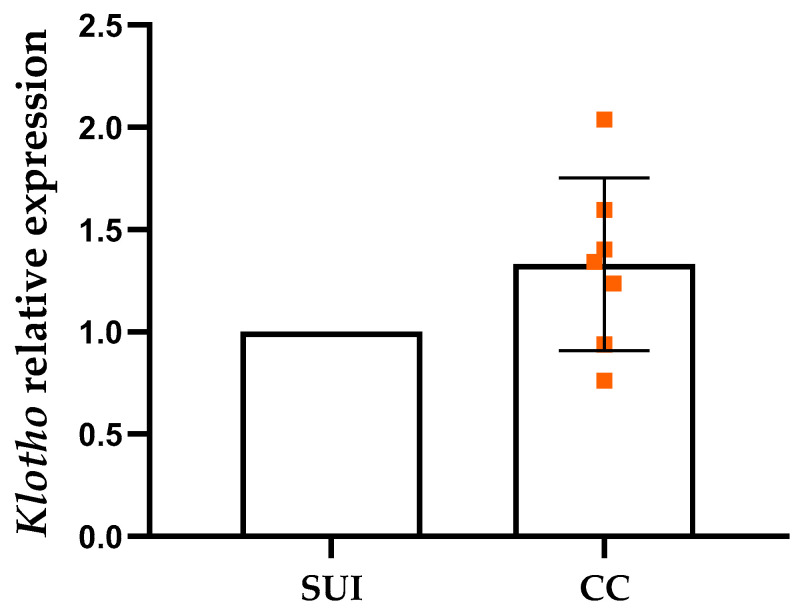
Analysis of *Klotho* in SUI fibroblasts after co-culture with DMSCs. Paracrine effect of DMSCs on SUI fibroblast co-cultured with on Transwell^®^ culture inserts. Fold changes in *Klotho* gene expression in SUI fibroblasts co-cultured with DMSCs (CC) relative to the corresponding SUI fibroblasts (SUI) (*n* = 7). Data are presented as the mean ± standard deviation (SD).

Tissue homeostasis is crucially influenced by Klotho, a longevity-associated protein involved in regulating cellular aging and tissue repair processes [65]. Our research indicates that *Klotho* expression is elevated in SUI fibroblasts when they are co-cultured with DMSCs compared to non-co-cultured SUI fibroblasts (Figure 8). The cytoprotective effects of Klotho are linked to the modulation of oxidative stress, inflammation, and cell cycle regulatory pathways, including the p53/p21 and Wnt/β-catenin signaling pathways [65]. Klotho deficiency in fibroblasts has been linked to cellular senescence and dysregulation of ECM remodeling, as evidenced by studies on pelvic floor disorders [66,67]. Recent clinical evidence indicates a correlation between the severity of SUI and serum Klotho levels [68]. According to data from the National Health and Nutrition Examination Survey (NHANES), middle-aged and older women with severe urinary incontinence had significantly lower serum levels of α-Klotho compared to continent controls [68]. The data on reduced Klotho expression in pelvic floor disorders indicate that it contributes to impaired tissue repair and dysregulation of the ECM. The increased *Klotho* expression following co-culture of SUI fibroblasts with DMSCs may reflect an effect of DMSCs contributing to improved regulatory mechanisms of the ECM and tissue homeostasis. A recent study in this line indicates that FGF21-mediated activation of cardiac FGFR1-β-Klotho induces cardiomyocyte secretion of CCL2/MCP-1, thereby mitigating inflammation in the diabetic heart [69]. Additionally, increased Klotho levels suppress the proliferation of interstitial fibroblasts and attenuate kidney fibrosis. Furthermore, Klotho has protective effects against inflammation and reduces fibrosis due to its ability to block TGF-β [70]. Therefore, Klotho acts as a systemic TGF-β inhibitor by targeting the TGF-β/Smad signaling pathways, influencing fibrosis and inflammation [70]. In our study, the *Klotho* elevations were associated with the protective effects through ECM remodeling and, in part, through reduced TGF-β levels in the SUI-DMSC co-culture.

**Figure 9:**
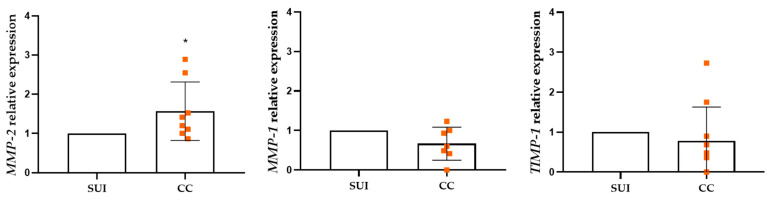
Analysis of matrix metalloproteases (MMPs) in SUI fibroblasts after co-culture with DMSCs. Paracrine effect of DMSCs on SUI fibroblast co-cultured with on Transwell^®^ culture inserts. Fold changes in the expression of matrix metalloprotease-2 (*MMP-2*) and -1 (*MMP-1*) genes, as well as tissue metalloprotease inhibitor-1 (*TIMP-1*) gene, in SUI fibroblasts co-cultured with DMSCs (CC) relative to the corresponding SUI fibroblast (SUI) (*n* = 7). Data are presented as the mean ± standard deviation (SD). * *p* < 0.05.

The matrix metalloproteases (MMPs) play a fundamental role in the physiological processes of tissue remodeling. However, their deregulated expression during senescence can contribute to the structural and functional deterioration of tissues. Matrix metalloproteinase-1 (MMP-1) and matrix metalloproteinase-2 (MMP-2) orchestrate ECM remodeling through collagen degradation, whereas tissue inhibitor of metalloproteinases-1 (TIMP-1) modulates proteolytic activity to maintain structural and cellular homeostasis. It is widely accepted that increased collagen breakdown is a pathological cause of SUI [71]. The results obtained here demonstrate that co-culture with DMSCs significantly increased *MMP-2* expression in SUI fibroblasts compared to cells without co-culture (Figure 9). In the case of *MMP-1*, DMSCs have been observed to decrease it in SUI cells, while *TIMP-1* remains unchanged or shows a slight decrease. However, these changes did not reach statistical significance, and therefore, they should be interpreted with caution. These results indicate that DMSCs may potentially restore certain molecular pathways associated with reduced cellular senescence.

Subsequent to tissue injury, ECM remodeling is a dynamic process that relies on a precise balance between matrix synthesis and proteolytic degradation, in which matrix metalloproteases play a central role [72]. In this context, MMP-2 functions as a key gelatinase by degrading basement membrane and ECM components, thereby facilitating matrix turnover required for tissue repair [73]. In wound healing, MMP-2 shows a delayed and localized expression pattern, primarily in fibroblasts, with peak levels occurring 8–12 weeks after injury. This finding indicates a predominant role during the remodeling phase rather than the early inflammatory response [74]. The activity of MMP-2 is subject to stringent regulation by tissue inhibitors of metalloproteases (TIMPs). Disruption of this regulatory balance can result in disorganized or chronic remodeling [73,75,76]. The collective evidence indicates that MMP-2 functions not only as a marker of tissue damage but also as an active and essential regulator of post-injury ECM reorganization [73,76].

The decrease in *MMP-1* gene expression observed in cells isolated from patients with SUI after co-culture with perinatal DMSCs can be interpreted as a potentially beneficial effect in the context of tissue regeneration. Although MMP-1 is necessary in the early stages of repair to facilitate controlled collagen degradation and cell migration [77,78,79], its elevated or sustained activity has been associated with excessive ECM degradation, loss of structural support, and poor healing in tissues subjected to chronic damage [80,81]. In this context, the reduction in *MMP-1* induced by perinatal DMSCs could contribute to preserving collagen integrity and promoting more stable and functional tissue remodeling, consistent with a more balanced regenerative environment [82]. These findings indicate that DMSCs have a regulatory effect on matrix remodeling, adjusting *MMP-1* expression to levels more suitable for regeneration. The reduction of MMP-1 could contribute to preserving the mechanical integrity of the tissue, while the increase in MMP-2 would allow the matrix plasticity necessary for functional repair. The observation that *TIMP-1* does not increase, but rather exhibits a slight decrease, suggests that DMSCs do not induce a global inhibition of proteolytic activity. Instead, they appear to promote a dynamic balance between matrix degradation and stabilization.

These results may suggest that DMSCs prevent excessive degradation and promote the remodeling necessary for regeneration [82,83]. This coordinated pattern of MMPs and TIMPs is consistent with a regulatory paracrine effect of DMSCs on the matrix microenvironment, particularly relevant in a chronically altered tissue such as the pelvic floor.

**Figure 10:**
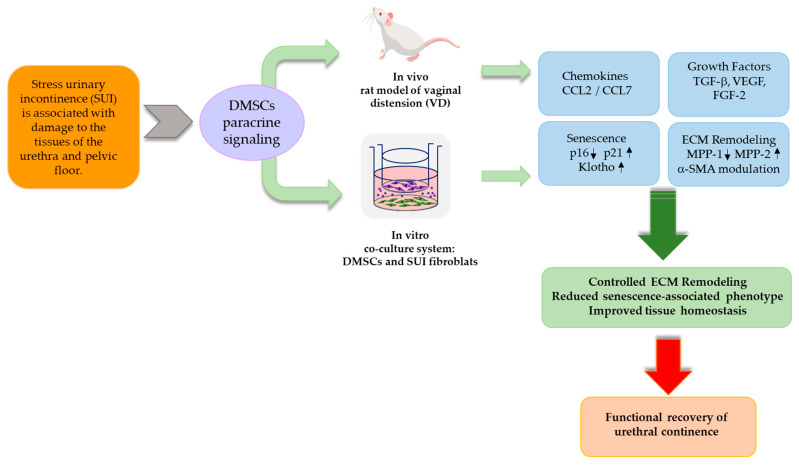
Proposed mechanisms by which decidua-derived mesenchymal stromal cells (DMSCs) may promote tissue repair and functional recovery following vaginal distension (VD). Stress urinary incontinence (SUI) is associated with damage to the urethral and pelvic floor tissues, typically resulting from childbirth-related mechanical trauma. This injury leads to hypoxia, inflammation, extracellular matrix (ECM) remodeling, cellular senescence, and impaired urethral function. Following periurethral administration, DMSCs exert paracrine effects that modulate multiple biological pathways involved in tissue repair. In the animal model, DMSC treatment was associated with modulation of chemokine signaling (CCL2 and CCL7), growth factor responses (TGF-β, VEGF, and FGF), and remodeling-associated markers (α-SMA), suggesting a more regulated progression through the repair process. In human SUI fibroblasts, DMSCs reduced p16 expression, increased Klotho expression, and altered matrix remodeling markers, decreasing MMP-1 while increasing MMP-2 expression. Collectively, these changes may contribute to a regenerative microenvironment that favors controlled ECM remodeling and improved recovery of urethral function, as reflected by improved leak point pressure (LPP). The results of molecular analyses provide a mechanistic understanding of the histological and functional improvements conferred by DMSCs after vaginal distension (VD), as described by us. However, the proposed mechanisms should be considered hypothetical, as they are based solely on gene expression analysis and require confirmation at the protein level. The direction of the arrow indicates the level of expression: ↑ increased expression; ↓ decreased expression.

To facilitate the interpretation of the findings from in vivo and in vitro experiments, Figure 10 presents a schematic model summarizing the proposed mechanisms of action of DMSCs. This model integrates functional, transcriptomic, and in vitro observations and illustrates how DMSC-mediated paracrine signaling can modulate pathways associated with senescence, responses to growth factors, chemokine signaling, and extracellular matrix remodeling. Although further validation at the protein and functional levels is necessary to fully substantiate these findings, the results generally indicate the generation of a regenerative microenvironment conducive to tissue repair and the restoration of urethral function. The molecular analyses presented in this work provide mechanistic insights into the histological and functional improvements previously described by our group.

## 3. Strengths and Limitations

The present study has several important strengths. First, it extends our previous work by providing a mechanistic characterization of the regenerative effects of decidua-derived mesenchymal stromal cells (DMSCs), a clinically relevant and readily available perinatal cell source, in a translational model of childbirth-related stress urinary incontinence (SUI). The study integrates complementary experimental approaches by combining a validated animal model with human SUI fibroblasts, thereby enhancing the translational relevance of the findings. A major strength is its integrative temporal design, which combines early molecular analyses in an independent one-week cohort with late molecular analyses performed on urethral tissues collected at 6 weeks from the same animals in which functional recovery and histological improvement had previously been demonstrated. This strategy enabled us to relate temporal changes in gene expression to established functional and structural outcomes, providing novel mechanistic insight into the biological processes associated with DMSC-mediated tissue repair. Furthermore, the simultaneous evaluation of molecular pathways involved in apoptosis, proliferation, inflammation, angiogenesis, extracellular matrix (ECM) remodeling, and cellular senescence offers a comprehensive overview of the regenerative response and supports the translational potential of DMSC therapy for stress urinary incontinence (SUI).

The study also has limitations. The vaginal distension (VD) model reproduces several key features of childbirth-related pelvic floor injury, such as ischemia, mechanical stretching, and transient urethral dysfunction. However, the model does not fully replicate the complexity of postpartum SUI in humans, which develops over several years and is influenced by multiple factors, including aging, hormonal changes, repeated pregnancies, and varying degrees of pudendal nerve injury. Therefore, caution should be exercised when extrapolating these findings directly to clinical practice. Another limitation of this study is the small number of animals in each experimental group. This may have reduced the statistical power to detect differences in some molecular parameters and contributed to the variability observed in the gene expression analyses. Therefore, these molecular findings should be interpreted as supporting evidence of the biological processes associated with tissue repair rather than as definitive mechanistic proof. Nevertheless, there is consistency between the functional improvement in urethral continence (LPP) and the trends observed in the analyzed molecular markers, which supports the biological relevance of the observed effects. Future studies with larger experimental cohorts and complementary molecular approaches are needed to validate these findings.

This study is primarily based on gene expression analyses. While the transcriptional changes observed in this study offer valuable insights into the biological pathways that may be modulated by DMSC treatment, it’s important to note that gene expression does not necessarily predict protein abundance or activity. Therefore, the present findings should be interpreted as evidence of modulation of biological pathways, rather than as direct confirmation of protein activity. Future studies involving protein quantification and functional analyses will be necessary to validate these molecular mechanisms.

DMSCs offer several advantages as a therapeutic platform for SUI. First, they are obtained from placental tissue that is routinely discarded after childbirth. This process circumvents the need for invasive extraction procedures and addresses ethical considerations. Second, DMSCs exhibit high proliferative capacity and immunomodulatory properties, along with low immunogenicity. Third, in contrast to autologous cell therapies, DMSCs can be manufactured as a ready-to-use allogeneic product, thereby facilitating standardization and wider clinical implementation. However, their allogeneic nature may also pose translational challenges, such as regulatory requirements and the need for further long-term immunocompatibility assessment.

## 4. Materials and Methods

### 4.1. Isolation and Culture of Mesenchymal Stromal Cells from the Decidua

Human placentas were collected from healthy mothers in the Department of Obstetrics and Gynecology after childbirth. The Ethics Committee of Hospital Universitario 12 de Octubre approved the collection, which was performed with written informed consent. The placental membranes were dissected and the cells obtained according to the well-established protocol [21]. In summary, the tissue was digested twice with 0.05% trypsin-EDTA (Gibco, Thermo Fisher Scientific, Waltham, MA, USA) for 30 min each time. The isolated cells were cultivated in Dulbecco’s Modified Eagle Medium (DMEM) containing 2 mM GlutaMAX and 0.1 mM sodium pyruvate (Gibco, Thermo Fisher Scientific, Waltham, MA, USA), further enriched with 1% nonessential amino acids (NEAA) (STEMCELL Technologies, Vancouver, BC, Canada), 1% penicillin/streptomycin (Gibco, Thermo Fisher Scientific, Waltham, MA, USA), 10% inactivated fetal bovine serum (FBS) (Biowest, Nuaillé, France), and 10 ng/mL epidermal growth factor (EGF) (Sigma-Aldrich, St. Louis, MO, USA). The cells were maintained at 37 °C in a controlled environment with 5% CO_2_ and 95% humidity and expanded in 6-well plates. The cells used in this study were characterized by flow cytometry and were found to be positive for the markers CD90, CD44, CD105, and CD73, and negative for the hematopoietic markers CD34 and CD45, as previously demonstrated [14,21,84,85,86]. These cells were termed decidua-derived mesenchymal stromal cells (DMSCs).

### 4.2. Animal Model of Stress Urinary Incontinence Due to Childbirth

Vaginal distension (VD) was induced in twelve 17-week-old virgin female Sprague-Dawley rats (300–350 g) to mimic maternal injuries during childbirth, according to our previously published method [14]. Briefly, isoflurane and ketamine-xylazine were used as anesthetics. After accommodating the vagina with lubricated bougie dilators, a modified 10-Fr Foley catheter was inserted and the balloon was inflated with 2 mL of PBS over half an hour, later increasing to 3 mL. The rats were positioned in the anti-Trendelenburg position, and the balloon was maintained for six hours. Post-procedure pain was managed with subcutaneous administration of buprenorphine. A Foley catheter was inserted into the vagina of the sham animals for six hours, though the balloon was not inflated. The sample size was determined a priori using G*Power software (version 3.1.9.7), which indicated that 5 to 7 animals were required per experimental group, assuming an alpha power of 0.05 and a beta power of 0.8. For the present study, six animals per group were selected, as this number met the estimated statistical requirements and was consistent with the 6-week experimental cohort previously described by our group [14]. One animal from each group died during the vaginal distension procedure and before tissue collection. Thus, the final analyses of the 1-week group included five animals in the VD and VD + DMSCs groups. The analyses of the 6-week group included six animals per group because the tissues had already been collected. A control group of three animals with no VD or DMSC treatment (CONTROL) was included in the 1-week study and two animals in the 6-week study.

### 4.3. DMSC Treatment

Following VD, the animals were randomly divided into two groups of five: one group received no treatment after VD, and the other group received treatment with DMSCs after injury (VD + DMSCs). The VD + DMSCs group received two doses of DMSCs resuspended in Hank’s balanced saline solution (HBSS) at a concentration of 2 × 10^6^ DMSCs per dose. The doses were administered one and three days after VD. The VD group received injections of the same volume of cell-free HBSS. The DMSC or HBSS injections were administered into the periurethral region via bilateral injections at the three and nine o’clock positions.

### 4.4. Leak Point Pressure (LPP) Measurement

The leak point pressure (LPP) measurement was performed while the animals were under anesthesia with inhaled isoflurane (5% for induction and 2% for maintenance), as previously described [14]. A PE-50 catheter was transurethrally inserted into the bladder. After emptying the bladder, its capacity was determined by filling it with PBS and methylene blue until leakage occurred. Three filling cycles were completed to calculate the average capacity. To measure the minimum pressure causing leakage (LPP), the bladder was filled to 50% capacity. A data acquisition device was connected to the catheter, and the intravesical pressure was recorded using AcqKnowledge software 5.0 (Biopac, Goleta, CA, USA). The LPP was determined by manually applying abdominal pressure [87]. Three measurements were carried out for each animal, and the mean pressure was calculated from these measurements. LPP was measured in millimeters of mercury (mmHg).

### 4.5. Isolation and Culture of Fibroblasts from Suburethral Tissue of Patients with Stress Urinary Incontinence (SUI)

This study examined eight suburethral tissue samples obtained from patients with SUI. Demographic and clinical characteristics of the patients are included in Table S1. The samples were obtained during a surgical procedure to insert a suburethral mesh sling in women with SUI at the Pelvic Floor Unit of the Department of Obstetrics and Gynecology at 12 de Octubre University Hospital. The Ethics Committee of the 12 de Octubre University Hospital approved the protocol, and informed consent was obtained from the patients. The cells were isolated by digesting the tissue enzymatically with 100 units/μL of collagenase type II (Gibco, Thermo Fisher Scientific, Waltham, MA, USA) in HBSS for 16 h. The digestion product was then centrifuged at 250 g for 10 min. The resulting pellet was then resuspended in complete DMEM for cell seeding. The cells were maintained at 37 °C with 5% CO_2_ throughout the expansion process.

### 4.6. Indirect DMSC-SUI Fibroblast Co-Culture System

To evaluate the effects of DMSCs on SUI fibroblasts in vitro, an indirect co-culture system was set up using Transwell inserts (Costar, Thermo Fisher Scientific, Waltham, MA, USA) with 0.4-μm-pore-size polycarbonate membranes. On the same day, fibroblasts were seeded in 6-well plates at a density of 3 × 10^5^ cells/well and DMSCs were seeded in the inserts at a density of 1.5 × 10^5^ cells/insert. The cell density and the ratio between the two cell types were selected to maintain consistency with our previously established co-culture conditions [14]. After 24 h, the inserts were transferred to the fibroblast-containing wells to initiate co-culture. The cells were then maintained under co-culture conditions for an additional 24 h prior to analysis.

### 4.7. Gene Expression Analysis

#### 4.7.1. RNA Isolation

Total RNA was extracted from the samples using the NZY Total RNA Isolation Kit (NZYTech, Ltd., Lisbon, Portugal) according to the manufacturer’s instructions. The concentration and purity of the RNA were assessed spectrophotometrically using a Nanodrop (Thermo Fisher Scientific, Waltham, MA, USA), and the extracted RNA was stored at −80 °C until further use.

#### 4.7.2. Retrotranscription

Complementary DNA (cDNA) was synthesized from 1 µg of total RNA using a High-Capacity cDNA Reverse Transcription Kit (Applied Biosystems, Foster City, CA, USA) according to the manufacturer’s protocol. The resulting cDNA was stored at −20 °C and used as a template for subsequent gene expression analyses.

#### 4.7.3. Quantitative Polymerase Chain Reaction (qPCR)

Gene expression analysis in urethral tissues was performed using quantitative PCR (qPCR) with TaqMan probes (Table S2), Fast TaqMan Master Mix (Applied Biosystems, Foster City, CA, USA), and a 7500 Fast Real-Time PCR System (Applied Biosystems, Foster City, CA, USA). PCR was performed under the following cycling conditions: an initial denaturation step at 95 °C for 2 min, followed by 40 cycles of denaturation at 95 °C for 3 s and annealing/extension at 60 °C for 30 s. TaqMan probes used in the study are included in Table S2. Target gene expression was normalized to TATA-box binding protein (TBP) and Glyceraldehyde 3-phosphate dehydrogenase (GAPDH) as reference genes by calculating the geometric mean of their expression levels. Relative gene expression levels were calculated using the 2^−ΔΔCt method, with the mean expression level of the control animals used as the calibrator.

For the study of SUI fibroblasts, quantitative PCR was performed using Fast SYBR Green Master Mix (Applied Biosystems, Foster City, CA, USA) and the 7500 Fast Real-Time PCR System (Applied Biosystems, Foster City, CA, USA). PCR was performed under the following cycling conditions: an initial denaturation step at 95 °C for 20 s, followed by 40 cycles of 95 °C for 3 s and annealing/extension at 60 °C for 30 s. Target gene expression was normalized to TBP as the reference gene. Relative gene expression levels were calculated using the 2^−ΔΔCt method. Gene expression in cocultured cells was compared with that in the corresponding non-cocultured cells. Primer sequences are listed in Table S3.

Klotho analysis in SUI fibroblasts was performed using Taqman technology with Fast TaqMan Master Mix (Applied Biosystems, Foster City, CA, USA) and a 7500 Fast Real-Time PCR System (Applied Biosystems, Foster City, CA, USA). The conditions of PCR were the same as those described for urethral gene expression. Target gene expression was normalized to TBP as the reference gene. Relative gene expression levels were calculated using the 2^−ΔΔCt method. Gene expression in cocultured cells was compared with that in the corresponding non-cocultured cells.

### 4.8. Statistical Analysis

Data were analyzed using GraphPad Prism software (version 8.0; GraphPad Software, San Diego, CA, USA). Data distribution was assessed using the Shapiro–Wilk normality test. Outliers were identified using the ROUT method (Q = 2%) implemented in GraphPad Prism and were excluded from the corresponding analyses. Statistical analyses were based on pre-specified hypothesis-driven pairwise comparisons to evaluate the therapeutic effect of DMSC treatment in animals subjected to vaginal distension. Therefore, no adjustment for multiple comparisons was applied. Differences between groups were analyzed using an unpaired Student’s t-test for normally distributed data or the Mann–Whitney U test for non-normally distributed data, as appropriate. A *p* value < 0.05 was considered statistically significant. Data are presented as mean ± standard deviation (SD).

## 5. Conclusions

Treatment with DMSCs has been shown to promote functional recovery in animals subjected to vaginal distension (VD), a model of childbirth-related damage. Functional improvements were evident at one and six weeks’ post-treatment. Transcriptomic analysis indicated that all animals after VD exhibited damage-repair responses, though only some recovered continence. The majority of displayed persistent changes in gene expression were linked to tissue remodeling, suggesting ongoing repair even six weeks after injury. While significant functional recovery differences were noted between treated and untreated groups, molecular differences were less pronounced, revealing individual variability reflective of women’s varying susceptibility to postpartum urinary incontinence and recovery processes. Gene analysis indicated that DMSCs influence repair dynamics, promoting earlier progression through the repair process without altering the repair process itself. At six weeks, untreated animals demonstrated sustained elevated growth factor levels, while treated animals exhibited a trend towards normalization, suggesting modulation of repair dynamics. However, the VD model has limitations due to its simplified representation of human childbirth, which results in a different type of injury and faster recovery, and may underestimate chronic urinary incontinence in humans. Another limitation of this study is the small number of animals included in each experimental group. Future studies should include a larger number of animals and combine the VD model with pudendal nerve injury to more accurately simulate postpartum incontinence.

The findings suggest that DMSCs have therapeutic potential in stress urinary incontinence (SUI) through paracrine effects that improve the regenerative environment by reducing senescence in suburethral fibroblasts and enhancing tissue dynamics, as demonstrated by changes in molecular markers like p16, p21, and Klotho. Our findings suggest that the role of DMSCs is to create a regenerative microenvironment that promotes tissue repair by modulating pathways associated with senescence and extracellular matrix remodeling.

The current findings offer insights into the molecular mechanisms that may underlie the previously observed functional and histological beneficial effects of DMSCs. Future studies should focus on validating the key pathways identified here at the protein level, characterizing the secretome and extracellular vesicles responsible for DMSC-mediated effects, and determining the persistence of these therapeutic responses in long-term experimental models. Furthermore, combining vaginal distension models with pudendal nerve injury could provide a more clinically relevant representation of postpartum stress urinary incontinence. These studies are essential for advancing the translational development of DMSC-based therapies for stress urinary incontinence.

## Data Availability

The raw data supporting the conclusions of this article will be made available by the authors on request.

## Supplementary Materials

The following supporting information can be downloaded at: https://www.mdpi.com/article/10.3390/ijms27156845/s1.

## Abbreviations

The following abbreviations are used in this manuscript:

SUI	Stress urinary incontinence
VD	Vaginal distension
DMSC	Decidua-derived mesenchymal stromal cells
LPP	Leak point pressure
ECM	Extracellular matrix
CCL2	Monocyte Chemoattractant Protein-1 (MCP-1)
CCL7	Monocyte Chemoattractant Protein-3 (MCP-3)
TGF-β	Transforming growth factor beta
VEGF	Vascular endothelial growth factor
FGF	Fibroblast growth factor
α-SMA	Smooth muscle alpha-actin
MMPs	Matrix metalloproteases
TIMP-1	Tissue inhibitor of metalloproteases 1
