# Supplementary material for: A Cell-Based Therapeutic Strategy for Stress Urinary Incontinence: Functional and Molecular Evidence from Decidua-Derived Mesenchymal Stromal Cells
Source: Int J Mol Sci. 2026 Jul 30;27(15):6845. doi: 10.3390/ijms27156845 (PMC13467398; doi:10.3390/ijms27156845)
Supplement: Supplementary file 1 [file ijms-27-06845-s001.zip › ijms-4445058-supplementary.pdf]

Table S1. Demographic and clinical characteristics of the patients with stress urinary incontinence (SUI) included in the study.

Patient	Age (years)	Weight (kg)	BMI (kg/m²)	Comorbidities	Pregnancies (n)
1	57	72	32	-	2
2	70	66	29.3	HT,DLP	4
3	45	50	18.8	-	1
4	62	76	30.8	-	1
5	70	64	28.1	HT,DLP	2
6	51	85	32.4	-	4
7	54	63	26.2	-	4
8	52	95	34.8	-	4

HT (Hypertension) and DLP (Dyslipidemia)

Table S2. TaqMan probes used in the study

Gene	Taqman probe
Rat *Tbp*	Rn01455646_m1
Rat *Gapdh*	Rn01775763_g1
Rat *Bcl-2*	Rn99999125_m1
Rat *Bax*	Rn01480161_g1
Rat *Ki-67*	Rn01451446_m1
Rat *Ccl-2*	Rn00580555_m1
Rat *Ccl-7*	Rn01467286_m1
Rat *Tgf-β*	Rn00572010_m1
Rat *Vegf*	Rn01511602_m1
Rat *Fgf*	Rn00570809_m1
Rat α–SMA (*Acta2*)	Rn01759928_g1
Human *Klotho*	Hs00934627_m1
Human *TBP*	Hs00427620_m1

Table S3. Primer sequences for human gene expression analysis

Gene	Forward Primer (5'–3')	Reverse Primer (5'–3')	Amplicon Size (bp
TBP	TGCACAGGAGCCAAGAGTGAA	CACATCACAGCTCCCCACCA	132
MMP-1	AGAGCAGATGTGGACCATGC	TTGTCCCGATGATCTCCCCT	134
MMP-2	CCTGAGGGCGCTCTGTCTC	GAAGGTGTTCAGGTATTGCACTG	133
TIMP-1	ATTCCGACCTCGTCACAGG	GCATCCCCTAAGGCTTGGAA	124
CDKN2A/p16	GAAGGTCCCTCAGACATCCCC	CCCTCTAGGACCTTCGGTGAC	94
CDKN1A/p21	GACTCTCAGGGTCGAAAACGG	CTTCCTCTTGGAGAAGATCAGCC	84
